# Prediction of Weight Loss to Decrease the Risk for Type 2 Diabetes Using Multidimensional Data in Filipino Americans: Secondary Analysis

**DOI:** 10.2196/44018

**Published:** 2023-04-11

**Authors:** Lisa Chang, Yoshimi Fukuoka, Bradley E Aouizerat, Li Zhang, Elena Flowers

**Affiliations:** 1 Department of Physiological Nursing University of California, San Francisco San Francisco, CA United States; 2 Keck Graduate Institute Claremont, CA United States; 3 Bluestone Center for Clinical Research New York University New York, NY United States; 4 Department of Oral and Maxillofacial Surgery New York University New York, NY United States; 5 Department of Epidemiology and Biostatistics University of California, San Francisco San Francisco, CA United States; 6 Department of Medicine University of California San Francisco San Francisco, CA United States; 7 Institute for Human Genetics University of California San Francisco San Francisco, CA United States

**Keywords:** type 2 diabetes, obesity, weight loss, feature selection, classification, transcriptomics

## Abstract

**Background:**

Type 2 diabetes (T2D) has an immense disease burden, affecting millions of people worldwide and costing billions of dollars in treatment. As T2D is a multifactorial disease with both genetic and nongenetic influences, accurate risk assessments for patients are difficult to perform. Machine learning has served as a useful tool in T2D risk prediction, as it can analyze and detect patterns in large and complex data sets like that of RNA sequencing. However, before machine learning can be implemented, feature selection is a necessary step to reduce the dimensionality in high-dimensional data and optimize modeling results. Different combinations of feature selection methods and machine learning models have been used in studies reporting disease predictions and classifications with high accuracy.

**Objective:**

The purpose of this study was to assess the use of feature selection and classification approaches that integrate different data types to predict weight loss for the prevention of T2D.

**Methods:**

The data of 56 participants (ie, demographic and clinical factors, dietary scores, step counts, and transcriptomics) were obtained from a previously completed randomized clinical trial adaptation of the Diabetes Prevention Program study. Feature selection methods were used to select for subsets of transcripts to be used in the selected classification approaches: support vector machine, logistic regression, decision trees, random forest, and extremely randomized decision trees (extra-trees). Data types were included in different classification approaches in an additive manner to assess model performance for the prediction of weight loss.

**Results:**

Average waist and hip circumference were found to be different between those who exhibited weight loss and those who did not exhibit weight loss (*P*=.02 and *P*=.04, respectively). The incorporation of dietary and step count data did not improve modeling performance compared to classifiers that included only demographic and clinical data. Optimal subsets of transcripts identified through feature selection yielded higher prediction accuracy than when all available transcripts were included. After comparison of different feature selection methods and classifiers, DESeq2 as a feature selection method and an extra-trees classifier with and without ensemble learning provided the most optimal results, as defined by differences in training and testing accuracy, cross-validated area under the curve, and other factors. We identified 5 genes in two or more of the feature selection subsets (ie, CDP-diacylglycerol-inositol 3-phosphatidyltransferase [*CDIPT*], mannose receptor C type 2 [*MRC2*], PAT1 homolog 2 [*PATL2*], regulatory factor X-associated ankyrin containing protein [*RFXANK*], and small ubiquitin like modifier 3 [*SUMO3*]).

**Conclusions:**

Our results suggest that the inclusion of transcriptomic data in classification approaches for prediction has the potential to improve weight loss prediction models. Identification of which individuals are likely to respond to interventions for weight loss may help to prevent incident T2D. Out of the 5 genes identified as optimal predictors, 3 (ie, *CDIPT*, *MRC2*, and *SUMO3*) have been previously shown to be associated with T2D or obesity.

**Trial Registration:**

ClinicalTrials.gov NCT02278939; https://clinicaltrials.gov/ct2/show/NCT02278939

## Introduction

### Background

Type 2 diabetes (T2D) is a metabolic disorder characterized by high blood glucose levels due to impaired insulin secretion or insulin resistance. T2D is one of three types of diabetes, which also includes gestational diabetes and type 1 diabetes; however, T2D accounts for 90%-95% of diabetes cases in the United States [1]. According to the Centers for Disease Control and Prevention, an estimated 88 million Americans have prediabetes and more than 34 million Americans have T2D [2]. In 2017, the United States spent US $327 billion on diabetes, with US $9601 spent on each individual with T2D [3]. The number of diabetes cases continues to increase and is expected to reach 693 million worldwide by the year 2045 [4].

A number of behavioral factors can alter the risk of developing T2D. Obesity is one of the leading T2D risk factors, as increased adipose tissue mass can lead to impaired insulin secretion or insulin resistance [5]. Diets high in saturated fats, refined grains, and sugar-sweetened beverages increase the risks of obesity and T2D [6]. Cultural and societal influences on diet may put certain populations and groups at higher risk of T2D. For example, certain racial and ethnic groups, including Filipino Americans, have been found to be more susceptible to developing T2D, with an estimated 2.5-fold higher T2D incidence compared to White adults [7]. Filipino American diets include a mix of carbohydrates and proteins like rice, vegetables, and meat [7]. These diets are associated with an overall increase in caloric and fat intake compared to the historical diets of Filipinos living in the Philippines [7]. In addition to the direct impact of evolving dietary patterns and cultural and social influences, evidence suggests there could be interactions with underlying ancestral genetic characteristics that interact with behavioral factors to increase risk [8].

Tools to screen for the risk of T2D have been created by the American Diabetes Association [9-11]. These tools consider common demographic and clinical risk factors like obesity and family history of diabetes. Risk prediction models can incorporate multiple variables relevant to T2D, but current models exhibit unreliable risk prediction [12]. Accurate assessment of behavioral data related to obesity and risk for T2D (ie, physical activity and diet) is challenging and can result in highly dimensional data sets that are difficult to analyze and interpret. Genome-wide association studies have identified a number of genes and single nucleotide polymorphisms that are significantly associated with T2D. Polygenic risk scores that include genetic variants known to be associated with T2D have been developed. However, the addition of these risk scores to models that include demographic (eg, family history) and clinical (eg, obesity) characteristics fails to provide a sufficiently accurate prediction of risk [13].

Interactions between the behavioral and genetic factors that contribute to the etiology of T2D make it a difficult condition to prevent and treat. In contrast to genetic information, assessment of the transcriptome, or the full set of expressed genes at a given moment in time within a specific tissue type from an individual, may provide insights about how an individual is responding to behavioral factors in the context of their underlying genetic characteristics. Transcriptome profiles change over time, including in response to changes in behavioral patterns. Because of this dynamic activity, the transcriptome may be a more useful means of assessing the combined impact of behavioral and genetic risk factors. However, as with physical activity and dietary data, transcriptomic data sets are highly dimensional and can be challenging to analyze and interpret.

### Prior Work

To address the challenge of complex and high-dimensional data sets, methods for optimal feature selection and machine learning algorithms have been developed [14]. Feature selection is a method that is employed to reduce the dimensionality of large data sets like transcriptomic data in order to capture the most relevant variables for outcome prediction. Machine learning algorithms include different types of classification approaches that use automated processes to discover patterns within large complex data sets to predict clinical outcomes [14]. Previous studies employed different classifiers in the prediction of the risk for T2D, using factors like BMI, blood pressure, age, and expression of long noncoding ribonucleic acid (lncRNA) [15]. When assessing lncRNA expression, the authors found that logistic regression and support vector machine (SVM) had the highest accuracy for predicting T2D [15]. Moreover, some classifiers performed better on specific data sets than others in a study that included 58 predictor variables to predict the outcome of fasting blood glucose [16]. The model that performed the best was also dependent on the observed metric score and the amount of available data [16]. The limitations of both studies were a small sample size, which may prevent accurate representation of the population, and limited generalizability, given the study sample characteristics. Additional studies that include individuals at the greatest risk for T2D based on social and biological characteristics are needed.

### Goal of This Study

The group of Filipino Americans is an example of an ethnic group at high risk for T2D, which has not been previously well represented in clinical research studies. The purpose of this study was to evaluate weight loss in response to a behavioral intervention tested in a previously completed clinical trial that included Filipino Americans. We integrated demographic and clinical data with behavioral and transcriptomic data to evaluate whether we could optimize the prediction of weight loss. We also identified the optimal transcriptomic features and determined their potential for mechanistic relationships with weight loss and the risk for T2D.

## Methods

### Study Participants

The data used in this secondary analysis were obtained from the Fit and Trim (F&T) Diabetes Prevention Program (DPP) study (ClinicalTrials.gov NCT02278939). This randomized, waitlisted, controlled trial was designed to assess the feasibility and acceptability of a DPP-based intervention in overweight Filipino Americans at risk for T2D. The goal of the intervention was to achieve 5% weight loss over 3 months. A total of 67 participants were recruited in the San Francisco area. The inclusion criteria were as follows: (1) self-identifying as Filipino American, (2) BMI >23 kg/m^2^, (3) age >24 years, (4) diabetes risk test score >5 points [17], (5) fasting plasma glucose level of 100-125 mg/dL, (6) hemoglobin A_1c_ (HbA_1c_) >5.6% or oral glucose tolerance test (OGTT) result of 140-200 mg/dL, (7) considered physically inactive based on the Brief Physical Activity Recall Questionnaire [18], (8) no cognitive impairment based on the Mini-Cog test [19], and (9) able to speak English. The exclusion criteria were as follows: (1) fasting blood glucose level >126 mg/dL, (2) OGTT result >200 mg/dL, (3) HbA_1c_ >7.0%, (4) glucose metabolism–associated disease, (5) thyroid disease that has been suboptimally treated, (6) special exercise program requirements, (7) current participation in a lifestyle modification program, (8) traveling outside the United States during the study period, (9) known eating disorders, (10) plans to have a gastric bypass surgery, (11) current pregnancy or delivery 6 months prior, (12) severe hearing or speech problems, and (13) use of antibiotics, antituberculosis agents (except tuberculosis prophylaxis), or prescription weight-loss drugs.

Demographic data were collected using a standardized questionnaire by trained study personnel. Blood pressure, waist and hip circumference, height, and weight were also collected by trained study personnel at each study visit. Blood was collected by venipuncture by trained study personnel at the enrollment visit following a 12-hour fast.

### Ethics Approval

This study was approved by the University of California, San Francisco Institutional Review Board (approval number: 19-29707), and participant consent was obtained before the start of the study.

### Behavioral Data

At enrollment, the Beverage Intake Questionnaire (BEVQ-15) and Fat-Related Diet Habit Questionnaire were used to assess dietary habits [20,21]. Participants were asked to wear a Fitbit Zip activity tracker for at least 10 hours per day to measure step count. The average daily step count over the last 4 weeks of the intervention period was used to characterize physical activity in prediction models.

### Study Design

Participants were randomized into one of two groups, which determined when they received the intervention. Regardless of which group they were placed in, all participants wore a Fitbit Zip device for the entire 6-month duration of the study to track and record daily step count. Those in the immediate group received a culturally tailored intervention and had access to a Facebook support group during the first 3 months (months 0-3) of the study. Those in the waitlist group received the intervention and had access to the support group during the last 3 months (months 3-6) of the study. For the study described in this manuscript, the 2 groups were “stacked” such that all data were analyzed simultaneously, with month 0 considered as baseline for the immediate group and month 3 considered as baseline for the waitlist group. Month 3 was considered as the final timepoint for weight loss in the immediate group, and month 6 was considered as the final timepoint for weight loss in the waitlist group.

### Molecular Data Collection

Blood was collected in PAXgene vacutainers (Qiagen) containing reagents to lyse cells and stabilize RNA molecules according to the standard protocol. Vacutainers were stored at −80 °C until RNA isolation was completed using the PAXgene blood RNAeasy kit (Qiagen) according to the standard protocol.

Library preparation and sequencing were performed by the University of California, Davis DNA Technologies and Expression Analysis Core Laboratory. Barcoded 3′-Tag-Seq libraries were prepared using the QuantSeq FWD kit (Lexogen) for multiplexed sequencing according to the recommendations of the manufacturer. The fragment size distribution of the libraries was verified via microcapillary gel electrophoresis on a Bioanalyzer 2100 system (Agilent). The libraries were quantified by fluorometry on a Qubit instrument (LifeTechnologies) and pooled in equimolar ratios. A total of 48 libraries were sequenced per lane on a HiSeq 4000 sequencer (Illumina) with single-end 100 base-pair reads.

### Data Preprocessing

Of the 67 participants in the parent trial, 11 were excluded for this analysis due to missing transcriptomic or step count data. Two of the remaining 56 participants had missing clinical data (ie, glucose, total cholesterol, triglycerides, low-density lipoprotein cholesterol, and high-density lipoprotein cholesterol), which were imputed using the mice package from R [22]. The parameter “pmm” or predictive mean matching was recommended and selected for imputation of continuous data. Sugar-sweetened beverage scores (calories and grams) were calculated based on a scoring guide, which included totaling up scores from sweetened fruit beverages, soft drinks, sweetened tea, tea or coffee with cream or sugar, and energy drinks [21]. The calculated fat score was the average of 5 factors (substitution, modify meat, avoid frying, replacement, and avoid fat) [20]. Changes in fat scores and sugar-sweetened beverage scores were then calculated between baseline and the end of the intervention for all participants. The average step count of the 4 weeks prior to completion of the intervention for each participant was used as a predictor variable. Due to the small sample size, 1 participant who had missing step count data for the previous 4 weeks was imputed with a mean of means involving all participants’ average step counts for the previous 4 weeks. Weight loss was defined as having a change in weight over 3 months of ≥5% of the baseline weight. Weight loss was then coded as “1” if there was ≥5% weight change and “0” otherwise for the outcome variable. The gene transcripts from the RNA-seq data were first filtered so that only those that appeared in 90% (51/56) of the samples and had ≥10 counts were retained. EdgeR was used to normalize the read counts for use in the feature selection methods, except the DESeq2 method [23,24].

### Statistical Analysis

Descriptive statistics were calculated for demographic and clinical characteristics overall and stratified by weight loss group, using the tableone package in Python [25]. The mean and SD were reported for continuous variables when the normality assumption held. Counts and percentages were reported for categorical variables. Two-group *t* tests were used to compare continuous variables between weight loss groups when the normality assumption held; otherwise, Wilcoxon rank sum tests were used. Chi-square tests were used for categorical variables. In addition to age, gender, and baseline weight, clinical and demographic variables with a *P* value <.05 based on a 2-sample *t* test were included in models that predicted weight loss. Statistical significance was declared based on a *P* value <.05. Through tableone default, Bonferroni correction was computed to account for multiple testing in Python.

### Feature Selection

For the transcriptomic data, the following 4 feature selection methods were evaluated: (1) Kolmogorov-Smirnov (K-S) test and correlation feature selection (CFS) [26], (2) correlation-based feature subset selection (CfsSubsetEval and BestFirst) [27], (3) differential gene expression using DESeq2 [23], and (4) modified Linear Forward Search & Maximum Relevance-Minimum Redundancy [28]. GreedyStepwise was applied as the search method for the K-S test and CFS method [26]. In addition, Maximum Relevance-Minimum Redundancy was modified to CfsSubsetEval, SubsetSizeForwardSelection, and Mutual Information and evaluated [28]. A combination of R, Python [29], and Waikato Environment for Knowledge Analysis (WEKA) [30], a data mining tool, was used to implement the feature selection methods.

The SVM classifier was used to determine the accuracy of the top 10, 9, 8, etc transcripts of each feature-selected subset. The accuracy of each size subset was compared for all the feature selection methods, and the top 5 transcripts had an optimal accuracy score. The top 5 transcripts of each feature selection method were then selected as predictors for the classifiers in the prediction of weight loss.

### Classifiers for Prediction

The Python library scikit-learn was used to run the following 5 supervised learning classification algorithms: (1) SVM, (2) logistic regression, (3) decision trees, (4) random forest, and (5) extremely randomized decision trees (extra-trees) [31]. Stratified 5-fold cross-validation was performed. Models were run with increasing complexity, starting with demographic and clinical characteristics and then adding behavioral characteristics, with the final addition of transcriptomic variables. After every model, parameter tuning was carried out. Parameter tuning was performed to select the optimal parameters for each algorithm, and then, each model was run again with the new set of parameters. Training and testing accuracy, cross-validated (CV) accuracy, area under the curve (AUC), CV AUC, precision, recall, and F1-scores were applied to assess and compare model performance.

Final risk models were run after incorporating all of the selected and statistically significant features from the different types of data available (ie, demographic, clinical, behavioral, and transcriptomic). These models were based on an ensemble method that used a bagging classifier to reduce variance by fitting classifiers on randomly generated subsets from the original data set and aggregating their individual predictions to form a final prediction [31]. SVM, logistic regression, decision trees, random forest, and extra-trees were all run with and without the bagging classifier. The same model performance metrics were applied to these final models.

## Results

Among the 56 participants, hip and waist circumference were found to be significantly different between the >5% weight loss and no weight loss groups, using a 2-sample *t* test (*P*=.02 and *P*=.04, respectively) (Table 1). The group that exhibited weight loss at the end of the intervention (n=25) had a smaller hip and waist circumference at baseline (Table 1). There was no difference between the immediate and waitlist groups at baseline (Table 1). More than half of the sample (31/56, 55%) identified as female (Table 1). The overall sample had a mean age of 43 (SD 13) years and was obese (mean BMI 30.1, SD 4.2 kg/m^2^) (Table 1).

The inclusion of all available transcripts that were normalized using edgeR (n=6088) in the SVM classifier resulted in overfitting, with a training accuracy and testing accuracy of 100% and 71%, respectively (Multimedia Appendix 1). Identification of the optimal subsets of transcripts using the 4-feature selection methods and filter criteria yielded varying numbers of transcripts and metric scores Multimedia Appendices 1-3). Overall, CV accuracy was higher when a feature selection method was applied than when using all 6088 transcripts. Using SVM, we determined that 5 was the optimal number of transcript features (Multimedia Appendix 1 and 2). On evaluating each of the subsets of 5 transcripts derived by different feature selection methods, DESeq2 had the smallest difference between the training and testing accuracy of 3%, with both an average CV accuracy and CV AUC of 83% (Multimedia Appendix 3). CfsSubsetEval, BestFirst, and Random Forest Ranker, and K-S test, CfsSubsetEval, and GreedyStepwise reported both an average CV accuracy and CV AUC of ≥90% and a training and testing accuracy difference of ≥21% (Multimedia Appendix 1 and 2). CfsSubsetEval, SubsetForwardSelection, and Mutual Information also had an average CV accuracy and CV AUC of >80%, while there was a 14% difference between the training and testing accuracy (Multimedia Appendix 1).

To assess how different types of data perform in different classifiers, SVM, logistic regression, decision trees, random forest, and extra-trees were run with data types in an additive manner (Multimedia Appendix 4). When using the extra-trees algorithm, demographic and clinical data only (ie, age, gender, baseline weight [pounds], and waist and hip circumference [cm]) yielded model scores of 50%-60% for testing accuracy, average cross-validation, AUC, and CV AUC (Table 2). Testing accuracy did not improve with the addition of the dietary behavior scores, while the average CV accuracy and CV AUC scores increased slightly (Table 2). When step count data were included, the testing accuracy and AUC scores dropped to 41%, while the average CV accuracy and CV AUC scores rose to approximately 80% (Table 2).

The final risk prediction models included the demographic and clinical data, dietary scores, step counts, and transcript subsets selected by feature selection methods with and without an ensemble approach (Table 3; Multimedia Appendices 5-7). Feature selection using DESeq2 and an extra-trees model yielded the best results (Table 3). When considering all the model metric scores collectively, the extra-trees model both with and without an ensemble approach had the smallest difference between the training and testing accuracy of 14% and 3%, respectively (Table 3). The CV AUC scores for both approaches were greater than 90% (Table 3).

**Figure 1:**
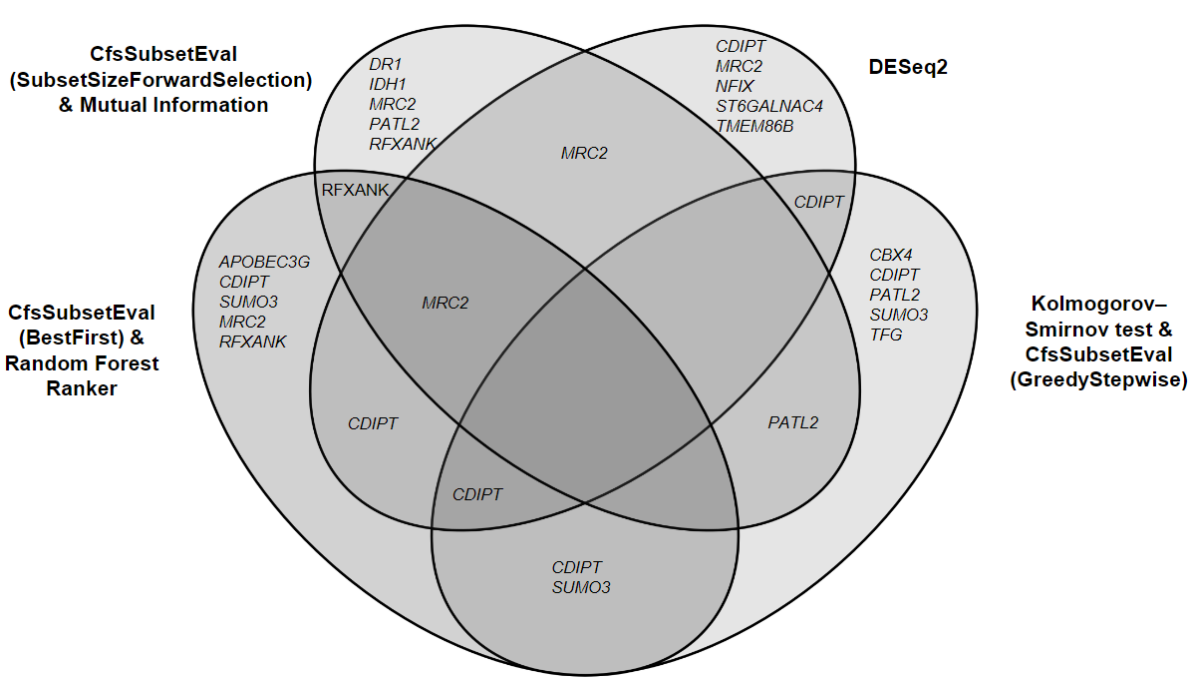
Venn diagram of overlapping and unique transcripts identified using 4 different feature selection methods. APOBEC3G: Apolipoprotein B MRNA Editing Enzyme Catalytic Subunit 3G; CBX4: Chromobox 4; CDIPT: CDP-Diacylglycerol-Inositol 3-Phosphatidyltransferase; CFS: correlation feature selection; DR1: Down-Regulator Of Transcription 1; IDH1: Isocitrate Dehydrogenase (NADP(+)) 1; MRC2: Mannose Receptor C Type 2; NFIX: Nuclear Factor I X; PATL2: PAT1 Homolog 2; RFXANK: Regulatory Factor X Associated Ankyrin Containing Protein; ST6GALNAC4: ST6 N-Acetylgalactosaminide Alpha-2,6-Sialyltransferase 4; SUMO3: Small Ubiquitin Like Modifier 3; TFG: Trafficking From ER To Golgi Regulator; TMEM86B: Transmembrane Protein 86B.

Five transcripts were selected as the optimal predictors using each feature selection approach (Figure 1). Five transcripts were found to overlap in at least two of the feature selection approaches (Figure 1), including mannose receptor C type 2 (*MRC2*), CDP-diacylglycerol-inositol 3-phosphatidyltransferase (*CDIPT*), regulatory factor X-associated ankyrin containing protein (*RFXANK*), small ubiquitin like modifier 3 (*SUMO3*), and PAT1 homolog 2 (*PATL2*).

**Table 1:** Demographic and clinical characteristics.

Variable			Overall (N=56)		No weight loss group (n=31)		>5% weight loss group (n=25)		*P* value
**Group, n (%) **									.40
	Immediate (0-3 months)	27 (48.2)		17 (54.8)		10 (40.0)			
	Waitlist (3-6 months)	29 (51.8)		14 (45.2)		15 (60.0)			
**Gender, n (%) **									.85
	Male	25 (44.6)		13 (41.9)		12 (48.0)			
	Female	31 (55.4)		18 (58.1)		13 (52.0)			
Age (years), mean (SD)			43 (13)		42 (12)		44 (13)		.58
BMI (kg/m^2^), mean (SD)			30.1 (4.2)		31.0 (5.0)		29.0 (2.6)		.06
Glucose level (mg/dL), mean (SD)			92 (10)		94 (10)		90 (9)		.17
Glucose change (mg/dL), mean (SD)			−2 (8)		−1 (8)		−3 (9)		.25
Total cholesterol level (mg/dL), mean (SD)			194 (31)		196 (33)		191 (30)		.52
Total cholesterol change (mg/dL), mean (SD)			−3 (25)		1 (22)		−8 (28)		.18
LDL^a^ cholesterol level (mg/dL), mean (SD)			115 (26)		118 (25)		112 (28)		.36
HDL^b^ cholesterol level (mg/dL), mean (SD)			52 (14)		52 (16)		54 (14)		.57
Weight (kg), mean (SD)			79.4 (15.4)		82.6 (17.2)		75.7 (12.2)		.07
Waist circumference (cm), mean (SD)			98 (10)		100 (11)		95 (7)		.04
Hip circumference (cm), mean (SD)			104 (9)		106 (11)		101 (5)		.02
Systolic blood pressure (mmHg), mean (SD)			126 (12)		128 (11)		125 (13)		.34
Diastolic blood pressure (mmHg), mean (SD)			78 (11)		79 (11)		76 (10)		.33

^a^LDL: low-density lipoprotein.

^b^HDL: high-density lipoprotein.

**Table 2:** Evaluation of extra-trees models.

Scoring metric	Model 1^a^	Model 2^b^	Model 3^c^	Model 4^d^	
				No ensemble	Ensemble
Training accuracy	0.85	0.97	0.97	0.85	0.90
Testing accuracy	0.59	0.53	0.41	0.82	0.76
Average CV^e^	0.56	0.61	0.85	0.83	0.77
AUC^f^	0.65	0.57	0.43	0.75	0.76
CV AUC	0.55	0.60	0.82	0.90	0.91
Precision	0.75	0.60	0.44	0.80	0.73
Recall	0.33	0.33	0.44	0.89	0.89
F1-score	0.46	0.43	0.44	0.84	0.80

^a^Model 1 included demographic (age and gender) and clinical (average waist and hip circumference, and baseline weight) characteristics.

^b^Model 2 included variables in Model 1 and dietary factors (fat-related diet habits summary score, and sugar-sweetened beverage average daily calorie and gram scores).

^c^Model 3 included variables in Model 2 and step count (average over the last 4 weeks).

^d^Model 4 included variables in Model 3 and the 5 most optimal transcripts selected by DESeq2.

^e^CV: cross-validated.

^f^AUC: area under the curve.

**Table 3:** Comparison of classifier results using all selected features.

Classifier and ensemble^a^		Training accuracy	Testing accuracy	Average CV^b^	AUC^c^	CV AUC	Precision^d^	Recall^d^	F1-score^d^
**SVM^e^**									
	Ensemble	0.79	0.47	0.80	0.50	0.92	0.50	0.56	0.53
	No ensemble	0.79	0.53	0.77	0.61	0.81	0.55	0.67	0.60
**Logistic regression**									
	Ensemble	0.90	0.41	0.85	0.46	0.85	0.44	0.44	0.44
	No ensemble	0.90	0.41	0.90	0.47	0.86	0.44	0.44	0.44
**Decision trees**									
	Ensemble	0.95	0.59	0.80	0.70	0.85	0.60	0.67	0.63
	No ensemble	0.95	0.53	0.85	0.65	0.84	0.60	0.33	0.43
**Random forest**									
	Ensemble	0.92	0.71	0.82	0.74	0.90	0.70	0.78	0.74
	No ensemble	0.95	0.71	0.82	0.72	0.87	0.70	0.78	0.74
**Extra-trees**									
	Ensemble	0.90	0.76	0.77	0.76	0.91	0.73	0.89	0.80
	No ensemble	0.85	0.82	0.83	0.75	0.90	0.80	0.89	0.84

^a^All models included demographic (age and gender), clinical (baseline weight, and waist and hip circumference), behavioral (dietary factors and step count), and transcript (5 most optimal predictors identified by DESeq2) features. An ensemble approach using a bagging classifier was assessed for each classifier.

^b^CV: cross-validated.

^c^AUC: area under the curve.

^d^Precision, recall, and F1-score for no weight loss (weight loss band=0).

^e^SVM: support vector machine.

## Discussion

### Summary of the Results

Analytic methods that incorporate both genetic and environmental factors to describe the risk for complex diseases like T2D may improve risk prediction. In this study, the use of demographic, clinical, and behavioral data did not result in highly accurate prediction of weight loss for the prevention of T2D. Although there are well known associations of dietary components and physical activity with weight and risk for T2D, in our models, these variables did not improve risk prediction (Table 2). The F&T trial was a feasibility study, and it is possible that the dose of the intervention was not sufficient to achieve a significant association with weight loss or that the specific measures of dietary factors and physical activity were not optimal for the weight loss outcome. Another explanation could be that in this study sample of people who identified as Filipino, the impact of genetic risk was greater than the impact of behavioral factors. The addition of gene transcripts into the models improved the prediction accuracy, but only when a subset of transcripts identified by feature selection was applied. Feature selection using DESeq2 reported the most optimal results when applied to an extra-trees model. A bagging classifier, the selected ensemble learning approach, also improved the AUC and CV AUC scores.

### DESeq2 Applied to Studies of T2D

Based on metrics for model performance, DESeq2 was found to be the best feature selection method for the data set in this study when the features were analyzed using an extra-trees model [23]. The training and testing accuracy had the smallest difference compared to all models, suggesting overfitting of the data was minimized. In contrast, a perfect (100%) training accuracy or a large difference in training and testing accuracy indicated possible overfitting in some of the observed models. DESeq2 is a popular R package available for differential gene expression that considers fold changes and dispersion rates by estimating shrinkage and is a conservative approach to control for false positives [23]. Most studies that focused on associations between the transcriptome and T2D used DESeq2 to identify differentially expressed genes that may be dysregulated or potentially involved in the pathogenesis of T2D and related complications [32,33]. Saxena et al [33] applied DESeq2 to identify 2752 differentially expressed genes (*P*<.01; log fold change ±2) using RNA expression data obtained from femoral subcutaneous adipose tissue in Asian Indians with and without diabetes. Another study identified 184 differentially expressed genes (adjusted *P*<.05; fold change ±2) from a total of 58,037 transcribed genes from the skin of individuals with and without T2D [23]. As a feature selection method, DESeq2 has been used to identify genes associated with small-cell lung cancer and integrated with other feature selection methods like EdgeR and Limma + voom to identify a smaller subset of overlapping genes [34]. Although DESeq2 has not appeared in studies as a feature selection method on its own, it offers the potential to select for a smaller more relevant subset of genes for risk predictions.

### Ensemble Learning in Studies of T2D

The 2 best approaches in this analysis included a model that used a bagging classifier for the ensemble learning approach and a model that did not. In addition to bagging, other ensemble learning approaches have been used to predict the risk for T2D [35], including stacking and boosting, which have the goal to improve modeling and make more accurate predictions [35]. Kumari et al [36] found that the soft voting classifier produced the highest scores with a prediction accuracy of 79.05% in a study of diabetes in Pima Indians. In the same sample, another study reported the highest prediction accuracy of 93.1% using a stacking classifier [35]. Within the same data set, the stacking classifier outperformed the soft voting classifier in not only accuracy but also precision, recall, and F1-scores [35,36]. However, both studies had relatively higher scores when using ensemble learning algorithms compared to models without these [35,36]. Similarly, in another study focused on the prediction of diabetic retinopathy, high accuracy was observed when a previously developed feature selection method and an original stacking-based ensemble learning technique (XGBIBS and Sel-Stacking, respectively) were used [37]. Jian et al [38] also compared different classification approaches and ensemble methods to predict the risk factors for T2D. Although XGBoost had the best performance, other models like logistic regression and random forest had higher metric scores when classifying metabolic syndrome and hypertension, respectively [38]. In the study described in this paper, the ensemble learning approach had higher AUC and CV AUC scores, but the model without the ensemble approach had higher testing and average CV accuracy. Although studies that focused on the prediction of the risk for T2D reported improved results with the inclusion of ensemble learning methods, our results suggest that ensemble learning will not always yield higher metric scores [39].

### Gene Functions/Pathways

Feature selection methods identified several genes that were found to be relevant to the weight loss outcome. In the subsets of 5 genes identified by feature selection, *CDIPT*, *MRC2*, *PATL2*, *RFXANK*, and *SUMO3* were found to overlap in at least two subsets. Some of these genes have known associations with the risk for T2D or obesity, while the function of others is less clear. Below is a review of evidence for associations between these genes and obesity or related risk factors.

Located on chromosome 16, *CDIPT* encodes for an enzyme that produces phospholipid phosphatidylinositol, which is a signaling molecule in lipid synthesis [40]. Previous studies linked abnormal *CDIPT* function to diseases like oral cancer or hepatic steatosis in zebrafish [40,41]. A *CDIPT* variant (hi559) was identified in zebrafish liver with upregulated endoplasmic reticulum stress markers [41]. This stress may be associated with insulin resistance in metabolic disorders like T2D and obesity [41]. Copy number variations (CNVs) in *CDIPT* have also been described in individuals with obesity or neurological disorders [42].

*MRC2* encodes for a receptor involved in extracellular matrix remodeling, cell migration, and invasion [43]. Upregulated *MRC2* expression has been detected in cancer tissues as well as in the peripheral blood of patients with diabetic nephropathy [43]. A simulation conducted to mimic glucose levels in T2D detected *MRC2* at high levels in mouse mesangial cells with high levels of glucose [43]. The study also found that knocking down *MRC2* using short interfering RNA (siRNA) affected the cell cycle and proliferation of mouse mesangial cells [43].

*PATL2* encodes for proteins that are predominantly expressed in oocytes and is responsible for inhibiting processes after transcription and translation [44]. *PATL2* mutations have mainly been associated with oocyte maturation and female infertility [45,46]. However, a study that looked at whole-genome expression found *PATL2* to be differentially expressed in obese and normal weight individuals with asthma compared to controls [47].

*RFXANK* encodes for a protein subunit of a larger complex that binds to major histocompatibility complex class II (MHCII) genes [48,49]. MHCII components are required for the adaptive immune response in which dysfunctions are associated with immunodeficiency disorders [49]. *RFXANK* mutations are prevalent in bare lymphocyte syndrome (BLS) group B, an immunodeficiency disorder affecting CD4+ T and B cells [50]. However, *RFXANK* has not been associated with obesity or T2D in previous studies, though MHCII has been found to play a role in obesity [51], and our own prior studies have identified pathways related to inflammation and immunity as common themes in individuals at risk for T2D [52]. Deng et al [51] analyzed *RFXANK* between 7 obese women and 7 lean postmenopausal women but did not find the expression to be significantly different.

*SUMO3* is involved in the posttranslation modification of target proteins known as sumoylation [53]. *SUMO3* has been found to be involved in disorders like obesity and neurodegenerative disorders like Parkinson disease and amyotrophic lateral sclerosis [53-55]. In a study that looked at obese and normal weight participants, proteomic analysis identified *SUMO3* to be one of the top 10 differentially expressed genes between the 2 groups [55]. Using microarray-based comparative genomic hybridization, another study found deleted *SUMO3* in an identified CNV in a child with syndromic obesity [56].

Additional studies are needed to determine the potential functional implications of the identified genes for T2D and obesity. *CDIPT* and *SUMO3* have been found to be differentially expressed in obese individuals; however, the exact mechanisms are not known. Upregulation of *MRC2* has been observed in people with T2D, and further studies are needed to determine whether these genes may be potential therapeutic targets.

### Limitations

Some limitations of this study were the modest sample size and missing data for some of the participants, requiring imputation. We were not able to exactly replicate feature selection methods from previous studies that required specific software and coding packages. We did not identify an external data set for validation that contained the necessary combination of variables (ie, dietary, step count, and transcriptomic). Future studies with larger sample sizes may also need to implement recent technological advances in methods for the collection of dietary and physical activity data. Some of the genes identified in this study are not known to be associated with obesity or the risk for T2D, and further assessment of potential functional relationships is needed.

### Conclusion

This study assessed multiple domains of individual characteristics for the prediction of weight loss in Filipinos at risk for T2D. This is one of the only studies to integrate transcriptomic data with behavioral data, and to our knowledge, this is the only study to apply this approach in the high-risk Filipino population. We identified optimal tools for feature selection and classification approaches for risk prediction, with an accuracy as high as 90% in the prediction of weight loss. Five genes were identified by multiple feature selection methods, including those known to be associated with conditions related to the risk for T2D and T2D complications.

## Appendix

Multimedia Appendix 1 Support vector machine modeling scores for transcripts selected by CfsSubsetEval, BestFirst, and Random Forest Ranker.

Multimedia Appendix 2 Support vector machine modeling scores for transcripts selected by the Kolmogorov-Smirnov test, CfsSubsetEval, and GreedyStepwise.

Multimedia Appendix 3 Support vector machine modeling scores for transcripts selected by DESeq2.

Multimedia Appendix 4 Classifier metric scores for different models.

Multimedia Appendix 5 Classification results for all data (demographic, clinical, behavioral, and transcriptomic data) using CfsSubsetEval, BestFirst (bidirectional search), and Random Forest Ranker with and without an ensemble approach.

Multimedia Appendix 6 Classification results for all data (demographic, clinical, behavioral, and transcriptomic data) using SubsetSizeForwardSelection, CfsSubsetEval, and Mutual Information with and without an ensemble approach.

Multimedia Appendix 7 Classification results for all data (demographic, clinical, behavioral, and transcriptomic data) using the Kolmogorov-Smirnov test, CfsSubsetEval, and GreedyStepwise with and without an ensemble approach.

## Abbreviations

AUC: area under the curve
CDIPT: CDP-diacylglycerol-inositol 3-phosphatidyltransferase
CFS: correlation feature selection
CNV: copy number variation
CV: cross-validated
DPP: Diabetes Prevention Program
K-S: Kolmogorov-Smirnov
lncRNA: long noncoding ribonucleic acid
MHCII: major histocompatibility complex class II
MRC2: mannose receptor C type 2
OGTT: oral glucose tolerance test
PATL2: PAT1 homolog 2
RFXANK: regulatory factor X-associated ankyrin containing protein
SUMO3: small ubiquitin like modifier 3
SVM: support vector machine
T2D: type 2 diabetes
