# Supplementary material for: Prediction of Weight Loss to Decrease the Risk for Type 2 Diabetes Using Multidimensional Data in Filipino Americans: Secondary Analysis
Source: JMIR Diabetes. 2023 Apr 11;8:e44018. doi: 10.2196/44018 (PMC10131631; doi:10.2196/44018)
Supplement: Multimedia Appendix 1 [file diabetes_v8i1e44018_app1.docx]

**Multimedia Appendix 1.** Support vector machine modeling scores for transcripts selected by CfsSubsetEval, BestFirst, and Random Forest Ranker.

| Number of Transcripts | Method | Filter | Training Accuracy | Testing Accuracy | Average CV | AUC | CV AUC | Precision | Recall | F1-Score |
| --- | --- | --- | --- | --- | --- | --- | --- | --- | --- | --- |
| 6088 | None | None | 1.00 | 0.71 | 0.64 | 0.63 | 0.74 | 0.67 | 0.89 | 0.76 |
| 28 | CfsSubsetEval | BestFirst | 1.00 | 1.00 | 0.95 | 1.00 | 0.99 | 1.00 | 1.00 | 1.00 |
| 5 |  | BestFirst, &  Random Forest Ranker, Top 5 | 0.95 | 0.71 | 0.93 | 0.83 | 0.95 | 0.67 | 0.89 | 0.76 |
| 25 | CfsSubsetEval | SubsetSizeForwardSelection | 1.00 | 0.82 | 0.90 | 0.96 | 0.96 | 0.80 | 0.89 | 0.84 |
| 5 |  | SubsetSizeForwardSelection, Mutual Information>0.1 | 0.90 | 0.76 | 0.85 | 0.93 | 0.93 | 0.78 | 0.78 | 0.78 |

Bestfirst performs a bidirectional search.

AUC – area under the curve; CV – cross validated; SVM – support vector machine

Precision, Recall, and F1-Score is for no weight loss (Weight Loss Band = 0)
