# Supplementary material for: Prediction of Weight Loss to Decrease the Risk for Type 2 Diabetes Using Multidimensional Data in Filipino Americans: Secondary Analysis
Source: JMIR Diabetes. 2023 Apr 11;8:e44018. doi: 10.2196/44018 (PMC10131631; doi:10.2196/44018)
Supplement: Multimedia Appendix 2 [file diabetes_v8i1e44018_app2.docx]

**Multimedia Appendix 2.** Support vector machine modeling scores for transcripts selected by the Kolmogorov-Smirnov test, CfsSubsetEval, and GreedyStepwise.

| Number of Transcripts | Method | Filter | Training Accuracy | Testing Accuracy | Average CV | AUC | CV AUC | Precision | Recall | F1-Score |
| --- | --- | --- | --- | --- | --- | --- | --- | --- | --- | --- |
| 6088 | None | None | 1.00 | 0.71 | 0.64 | 0.63 | 0.74 | 0.67 | 0.89 | 0.76 |
| 618 | KS test: Adj *p*-value ≤ 0.05 | None | 1.00 | 0.71 | 0.61 | 0.76 | 0.70 | 0.70 | 0.78 | 0.74 |
| 23 | KS test: Adj *p*-value ≤ 0.05 & CfsSubsetEval | GreedyStepwise | 0.97 | 0.94 | 0.90 | 1.00 | 0.96 | 0.90 | 1.00 | 0.95 |
| 5 | KS test: Adj *p*-value ≤ 0.05 & CfsSubsetEval | GreedyStepwise, Top 5 | 0.92 | 0.71 | 0.90 | 0.83 | 0.95 | 0.67 | 0.89 | 0.76 |

AUC – area under the curve; CV – cross validated; KS test - Kolmogorov–Smirnov test, SVM- support vector machine

Precision, Recall, and F1-Score is for no weight loss (Weight Loss Band = 0)
