# Supplementary material for: Prediction of Weight Loss to Decrease the Risk for Type 2 Diabetes Using Multidimensional Data in Filipino Americans: Secondary Analysis
Source: JMIR Diabetes. 2023 Apr 11;8:e44018. doi: 10.2196/44018 (PMC10131631; doi:10.2196/44018)
Supplement: Multimedia Appendix 3 [file diabetes_v8i1e44018_app3.docx]

**Multimedia Appendix 3.** Support vector machine modeling scores for transcripts selected by DESeq2.

| Number of Transcripts | Filter | Training Accuracy | Testing Accuracy | Average CV | AUC | CV AUC | Precision | Recall | F1-Score |
| --- | --- | --- | --- | --- | --- | --- | --- | --- | --- |
| 6088 |  | 1.00 | 0.47 | 0.75 | 0.56 | 0.83 | 0.50 | 0.44 | 0.47 |
| 41 | Adj *p*-value ≤ 0.1 | 0.87 | 0.71 | 0.77 | 0.81 | 0.85 | 0.70 | 0.78 | 0.74 |
| 5 | Adj *p*-value ≤ 0.1, Top 5 | 0.79 | 0.76 | 0.83 | 0.79 | 0.83 | 0.78 | 0.78 | 0.78 |

AUC – area under the curve; CV – cross validated, SVM – support vector machine

Precision, Recall, and F1-Score is for no weight loss (Weight Loss Band = 0)
