# Supplementary material for: Prediction of Weight Loss to Decrease the Risk for Type 2 Diabetes Using Multidimensional Data in Filipino Americans: Secondary Analysis
Source: JMIR Diabetes. 2023 Apr 11;8:e44018. doi: 10.2196/44018 (PMC10131631; doi:10.2196/44018)
Supplement: Multimedia Appendix 4 [file diabetes_v8i1e44018_app4.docx]

**Multimedia Appendix 4.** Classifier metric scores for different models.

| Model | Classifiers | Training Accuracy | Testing Accuracy | Average CV | AUC | CV AUC | Precision | Recall | F1-Score |
| --- | --- | --- | --- | --- | --- | --- | --- | --- | --- |
| Model 1 | SVM | 0.62 | 0.53 | 0.54 | 0.49 | 0.49 | 0.57 | 0.44 | 0.50 |
|  | Logistic Regression | 0.62 | 0.59 | 0.59 | 0.45 | 0.65 | 0.75 | 0.33 | 0.46 |
|  | Decision Trees | 0.64 | 0.47 | 0.71 | 0.49 | 0.71 | 0.50 | 0.44 | 0.47 |
|  | Random Forest | 0.82 | 0.59 | 0.56 | 0.76 | 0.58 | 0.75 | 0.33 | 0.46 |
|  | Extra-Trees | 0.85 | 0.59 | 0.56 | 0.65 | 0.55 | 0.75 | 0.33 | 0.46 |
| Model 2 | SVM | 0.95 | 0.41 | 0.61 | 0.32 | 0.58 | 0.46 | 0.67 | 0.55 |
|  | Logistic Regression | 0.72 | 0.59 | 0.49 | 0.51 | 0.43 | 0.75 | 0.33 | 0.46 |
|  | Decision Trees | 0.90 | 0.65 | 0.74 | 0.58 | 0.74 | 0.71 | 0.56 | 0.63 |
|  | Random Forest | 0.90 | 0.53 | 0.61 | 0.54 | 0.63 | 0.60 | 0.33 | 0.43 |
|  | Extra-Trees | 0.97 | 0.53 | 0.61 | 0.57 | 0.60 | 0.60 | 0.33 | 0.43 |
| Model 3 | SVM | 0.74 | 0.47 | 0.74 | 0.50 | 0.92 | 0.50 | 0.67 | 0.57 |
|  | Logistic Regression | 0.90 | 0.41 | 0.90 | 0.47 | 0.86 | 0.44 | 0.44 | 0.44 |
|  | Decision Trees | 0.85 | 0.47 | 0.76 | 0.42 | 0.73 | 0.50 | 0.56 | 0.53 |
|  | Random Forest | 0.90 | 0.47 | 0.85 | 0.43 | 0.82 | 0.50 | 0.56 | 0.53 |
|  | Extra-Trees | 0.97 | 0.41 | 0.85 | 0.43 | 0.82 | 0.44 | 0.44 | 0.44 |

AUC – area under the curve; CV – cross validated; SVM – support vector machine

Precision, Recall, and F1-Score is for no weight loss (Weight Loss Band = 0)

Model 1 includes demographic and clinical characteristics (age, gender, baseline weight (pounds), waist and hip circumference (centimeters))

Model 2 includes all variables in Model 1 plus dietary factor scores (sugar sweetened beverage consumption, dietary fat habits)

Model 3 includes all variables in Model 2 plus mean daily step counts measured over the last 4 weeks of the intervention
