# Supplementary material for: Prediction of Weight Loss to Decrease the Risk for Type 2 Diabetes Using Multidimensional Data in Filipino Americans: Secondary Analysis
Source: JMIR Diabetes. 2023 Apr 11;8:e44018. doi: 10.2196/44018 (PMC10131631; doi:10.2196/44018)
Supplement: Multimedia Appendix 7 [file diabetes_v8i1e44018_app7.docx]

**Multimedia Appendix 7.** Classification results for all data (demographic, clinical, behavioral, and transcriptomic data) using the Kolmogorov-Smirnov test, CfsSubsetEval, and GreedyStepwise with and without an ensemble approach.

| Classifiers | Ensemble | Training Accuracy | Testing Accuracy | Average CV | AUC | CV AUC | Precision | Recall | F1-Score |
| --- | --- | --- | --- | --- | --- | --- | --- | --- | --- |
| SVM | Y | 0.97 | 0.71 | 0.87 | 0.81 | 0.93 | 0.75 | 0.67 | 0.71 |
|  | N | 1.00 | 0.71 | 0.87 | 0.82 | 0.96 | 0.75 | 0.67 | 0.71 |
| Logistic Regression | Y | 0.95 | 0.59 | 0.92 | 0.63 | 0.94 | 0.62 | 0.56 | 0.59 |
|  | N | 0.95 | 0.65 | 0.90 | 0.65 | 0.96 | 0.67 | 0.67 | 0.67 |
| Decision Trees | Y | 0.95 | 0.76 | 0.72 | 0.75 | 0.91 | 0.73 | 0.89 | 0.80 |
|  | N | 0.92 | 0.59 | 0.85 | 0.54 | 0.89 | 0.58 | 0.78 | 0.67 |
| Random Forest | Y | 1.00 | 0.71 | 0.82 | 0.78 | 0.96 | 0.67 | 0.89 | 0.76 |
|  | N | 1.00 | 0.65 | 0.85 | 0.71 | 0.97 | 0.62 | 0.89 | 0.73 |
| Extra Trees | Y | 0.97 | 0.76 | 0.74 | 0.78 | 0.94 | 0.78 | 0.89 | 0.80 |
|  | N | 0.95 | 0.65 | 0.88 | 0.76 | 0.91 | 0.62 | 0.89 | 0.73 |

AUC – area under the curve; CV – cross validated; SVM – support vector machine

Precision, Recall, and F1-Score is for no weight loss (Weight Loss Band = 0)
